# Polarity kinases that phosphorylate F-BAR protein Cdc15 have unique localization patterns during cytokinesis and contributions to preventing tip septation in *Schizosaccharomyces pombe*

**DOI:** 10.17912/micropub.biology.000965

**Published:** 2023-09-08

**Authors:** Maya G. Igarashi, Rahul Bhattacharjee, Alaina H. Willet, Kathleen L. Gould

**Affiliations:** 1 Department of Cell and Developmental Biology, Vanderbilt University School of Medicine, Nashville, TN, US; 2 Current address: Biophysical Sciences, University of Chicago, Chicago, IL, US; 3 Current address: Twist Bioscience, Quincy, MA, US

## Abstract

The *Schizosaccharomyces pombe* F-BAR protein, Cdc15, facilitates the linkage between the cytokinetic ring and the plasma membrane. Cdc15 is phosphorylated on many sites by four polarity kinases and this antagonizes membrane interaction. Dephosphorylation of Cdc15 during mitosis induces its phase separation, allowing oligomerization, membrane association, and protein partner binding. Here, using live cell imaging we examined whether spatial separation of Cdc15 from its four identified kinases potentially explains their diverse effects on tip septation and the mitotic Cdc15 phosphorylation state. We identified a correlation between kinase localization and their ability to antagonize Cdc15 cytokinetic ring and membrane localization.

**Figure 1. Analysis of S. pombe Pom1, Kin1, Shk1 and Pck1 localization and role in tip septation:**
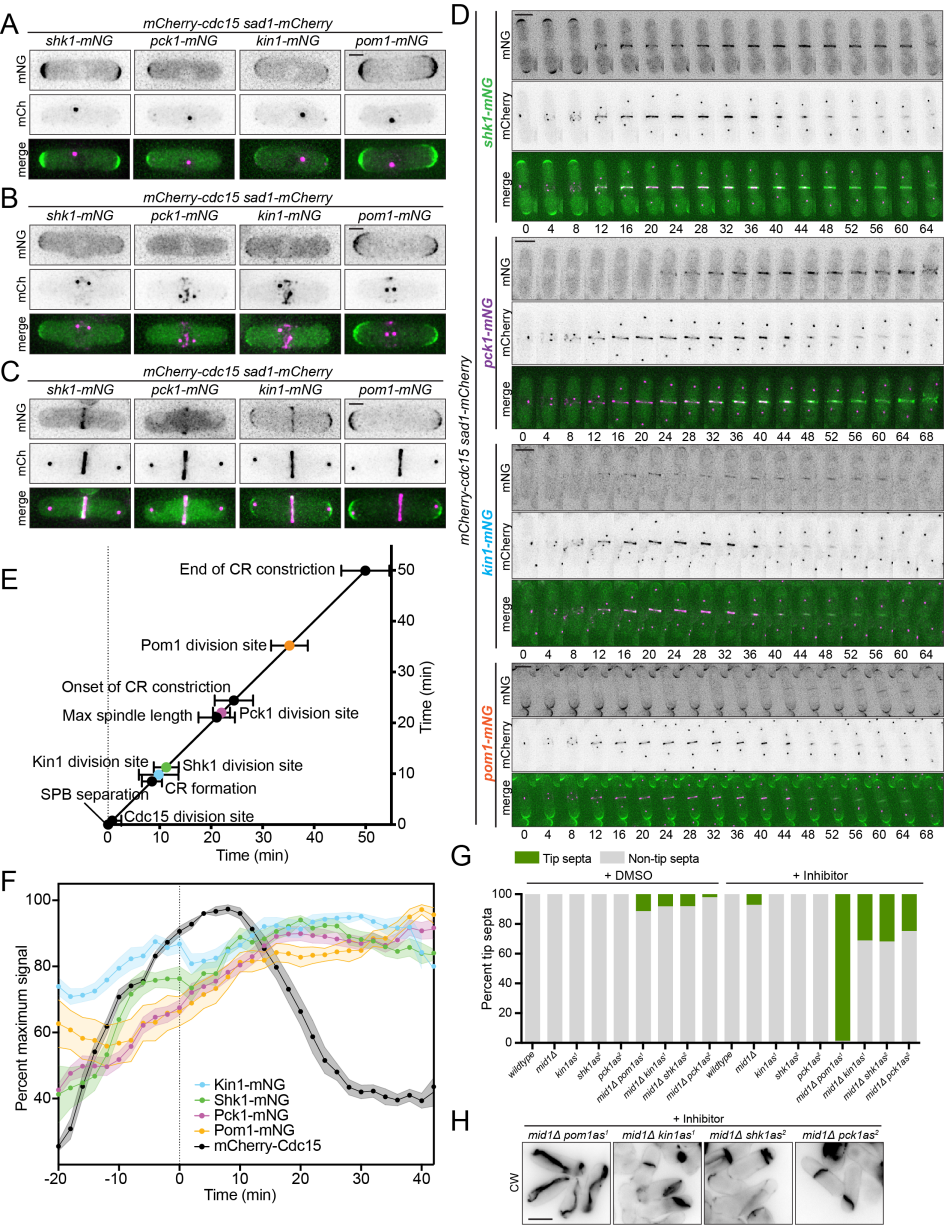
(A-C) Live-cell images of the localization of the indicated protein kinases tagged with the sequences encoding mNG at different cell division cycle stages indicated by Sad1-mCherry, a SPB marker, and mCherry-Cdc15, a CR marker. Scale bars, 2 µm. (D) Representative montages from live-cell time-lapse imaging of the indicated strains. Images were acquired every 2 minutes and images from every 4 minutes are shown. Numbers indicate minutes from SPB separation. Scale bars, 2 µm. (E) Timeline showing the detection of various proteins at the cell division site in relationship to cell division events. Time 0 was defined as SPB separation, and the mean time of detection of each protein ± SD is plotted. *n* ≥ 10 per strain. (F) Fluorescence intensity of mNG-tagged protein kinases at the cell division site plotted over time from movies as in E. Time 0 was defined as the time of maximum spindle length (dotted line). Fluorescence intensity measurements are reported as the percentage of the maximum cell division site intensity for each protein. Shaded areas represent SEM, *n* = 10 cells per strain. (G) The indicated strains were grown at 32°C, treated with appropriate inhibitor or DMSO and then fixed and stained with Calcofluor White (CW). Images of the indicated strains were quantified for the presence of tip septa, *n* ≥ 60 septated cells per strain. (H) Images of select indicated strains from H. Images are a single medial z-slice. Scale bar, 5 µm.

## Description

Cytokinesis is the terminal step in the cell division cycle that results in the physical separation of two new daughter cells. Many cells, including the yeast model organism *Schizosaccharomyces pombe,* build an actin- and myosin-based cytokinetic ring (CR) at the cell cortex that constricts to facilitate cell separation (Cheffings et al., 2016; Mangione and Gould, 2019; Pollard, 2010; Pollard and Wu, 2010) . In the case of *S. pombe* , the CR also guides the formation of a division septum (Perez et al., 2018; Willet et al., 2015) . One *S. pombe* CR component, the F-BAR Cdc15 , has been well characterized as an important link between the CR and the plasma membrane (PM) (Arasada and Pollard, 2014; Laporte et al., 2011; McDonald et al., 2015; Roberts-Galbraith et al., 2009; Roberts-Galbraith et al., 2010; Snider et al., 2020; Vjestica et al., 2008; Wachtler et al., 2006) .

Cdc15 is an essential protein for cytokinesis and is highly regulated via phosphorylation (Fankhauser et al., 1995; Roberts-Galbraith et al., 2010) . When dephosphorylated during mitosis, it is able to phase separate, oligomerize, bind membrane, and interact with other CR proteins to form a CR scaffold along the PM (Bhattacharjee et al., 2023) . Cdc15 is hyperphosphorylated during interphase by numerous protein kinases that play important roles in cell polarity: the DYRK kinase Pom1 , MARK/PAR-1 kinase Kin1 , protein kinase C Pck1 , and p21-activated kinase Pak1/ Shk1 /Orb2 (Bhattacharjee et al., 2020; Kettenbach et al., 2015; Lee et al., 2018; Magliozzi et al., 2020) . Hyperphosphorylation serves to inhibit membrane binding so that the majority of the phosphorylated forms are cytosolic, and this in turn inhibits CR assembly and cell septation (Roberts-Galbraith et al., 2010; Wachtler et al., 2006) .

Based on mutational analysis, it appears that no individual phosphorylation site or specific combination of phosphorylation sites inherently affects Cdc15 localization differently (Bhattacharjee et al., 2023) . Instead, it appears that the absolute level of phosphorylation governs Cdc15 function and each of the four defined protein kinases contribute different levels of phosphorylation (Bhattacharjee et al., 2023) . We hypothesized therefore that the Cdc15 kinases were either less active or spatially separated from Cdc15 at the time and/or place of CR formation so that phosphatases could be more effective in driving Cdc15 dephopshorylation.

To examine this possibility, each kinase was tagged with sequences encoding mNeonGreen (mNG) in *mCherry-cdc15* cells. These cells also produced Sad1-mCherry as a spindle pole body (SPB) marker to define cell cycle stages (Hagan and Yanagida, 1995) . In accord with previous reports (Bhatia et al., 2014; Gerganova et al., 2019; Hachet et al., 2011; Hachet and Simanis, 2008; Lee et al., 2018; Loo and Balasubramanian, 2008; Magliozzi et al., 2020) , all four kinases localized at cell tips during interphase when very little Cdc15 localized there ( Figure 1A ). During early mitosis when Cdc15 began to appear in nodes at the incipient division site, none of the four kinases were detected there ( Figure 1B ), consistent with the previous reports. In late anaphase, determined by maximum SPB separation and when the CR was fully formed, Shk1 , Kin1 , and Pck1 were detected at the CR with Cdc15 but Pom1 , the major Cdc15 kinase (Bhattacharjee et al., 2023) was detected only at cell tips ( Figure 1C ).

We examined the localization of the four Cdc15 kinases relative to Cdc15 with more precision using time-lapse imaging. Kin1 and Shk1 were lost from cell tips and appeared at the cell division site shortly after CR formation (Figures 1D-E). Pck1 arrived at the CR later than Kin1 or Shk1 , right before the onset of CR constriction (Figures 1D-E). Finally, Pom1 appeared at the division site during CR constriction and septation (Figures 1D-E). The co-localization of Cdc15 with three of its kinases during CR formation when Cdc15 is most hypophosphorylated (Fankhauser et al., 1995; Roberts-Galbraith et al., 2010) suggests that they may have lower activity or be out-competed by phosphatase action at this stage. However, if the kinases are active towards Cdc15 at this time, it could explain previous fluorescence recovery after photobleaching results, which demonstrated that a portion of Cdc15 is highly dynamic in the CR (Clifford et al., 2008; Kamnev et al., 2021; McDonald et al., 2015; Roberts-Galbraith et al., 2010) **.** Further, the accumulation of all four Cdc15 kinases at the CR during its constriction may aid in disassembling Cdc15 from the CR.

Tip-localized Pom1 plays a major role in preventing off-center septation and this role becomes essential in the absence of the positive cue for medial division, Mid1 (Chang et al., 1996; Huang et al., 2007; Rincon and Paoletti, 2016; Sohrmann et al., 1996) . The localization of Pom1 to cell tips during CR formation is consistent with its role in antagonizing septation there partly through Cdc15 phosphorylation (Bhatia et al., 2014; Bhattacharjee et al., 2020; Celton-Morizur et al., 2006; Martin and Berthelot-Grosjean, 2009; Padte et al., 2006) . Shk1 also plays a role in preventing tip septation when the positive cue for medial septation, Mid1 , is missing (Magliozzi et al., 2020) , also consistent with its detection at tips during CR formation. Because we observed some Kin1 and Pck1 at cell tips during CR formation, we tested if they, too, play a role in antagonizing tip septation in the absence of Mid1 . To inhibit the four kinases, we used the ATP analogs 3MB-PP1 or 3BrB-PP1 and strains sensitive to them, *shk1 ^as2^* (M460A) (Cipak et al., 2011) , *pom1 ^as1^* (T778G) (Padte et al., 2006) , *kin1 ^as1^* (F220G) (Cadou et al., 2010) , and *pck1 ^as2^* (M744G) (Bohnert et al., 2020) as previously described (Bhattacharjee et al., 2023) . Interestingly, the percentage of tip septa increased when either Kin1 ^as1^ , Shk1 ^as2^ , or Pck1 ^as2^ were inhibited in *mid1Δ* cells but not nearly to the same extent as observed when Pom1 ^as1^ was inhibited ( Figure 1G -H). Thus, while Shk1 , Kin1 , and Pck1 contribute to preventing tip septation, they do not play a significant role as compared to Pom1 , correlating with their diminished tip localization during CR formation.

## Methods

Yeast methods

*S. pombe* strains were grown in yeast extract (YE) and standard *S. pombe* mating, sporulation, and tetrad dissection techniques were used to construct new strains (Moreno et al., 1991) . Genes encoding protein kinases were tagged by inserting sequencing encoding mNG at the 3’end of the coding sequence with either kanMX6 or hphMX6 as selectable markers as described (Bahler et al., 1998; Wach et al., 1994) . Correct tagging was confirmed by whole-cell PCR.

Tip septa quantification

To inhibit Pom1 ^as1^ and Kin1 ^as1^ in vivo, cells were grown in YE at 32°C to mid-log phase and treated with 4-amino-1-tert-butyl-3-(3-methylbenzyl)pyrazolo[3,4-3]pyrimidine (3MB-PP1) (Toronto Research Chemical; A602960 or Cayman Chemical; 17860) at a final concentration of 15 µM for 30 minutes. Pck1 ^as2^ and Shk1 ^as2^ were inhibited with 30 µM 3-[(3-Bromophenyl) methyl]-1-(1,1-dimethylethyl)-1H-pyrazolo[3,4-d]pyrimidin-4-amine 4-amino-1-tert-butyl-3-(3-bromobenzyl)pyrazolo[3,4-d]pyrimidine (3BrB-PP1) (Abcam; ab143756) for 30 minutes. Both inhibitors (3MB-PP1 and 3BrB-PP1) were used at the above-mentioned concentrations to inhibit combinations of analog-sensitive kinase mutants. As control, cells were grown in equal volume of DMSO (Sigma; D2650). Yeast cells were fixed by adding ice-cold 70% ethanol while vortexing and then incubating at 4°C for at least 15 minutes. Cells were fixed at a ratio of 0.5 OD cells per 1 ml of 70% ethanol. Cells were washed once with phosphate-buffered saline (PBS), pH 7.5, and then resuspended in 20 µl of 50 µg/ml Calcofluor White (Sigma; 18909) and incubated at room temperature for 5 minutes. Then, cells were washed once with PBS and imaged immediately, as described (Bhattacharjee et al., 2020) .

Microscopy and image analysis

Yeast for live-cell imaging were grown at 25°C. Live-cell and fixed-cell imaging was performed on log-phase cells at 25°C using a Personal DeltaVision (Leica Microsystems) that includes a microscope (IX71; Olympus), 60 × NA 1.42 Plan Apochromat and 100 × NA 1.40 U Plan S Apochromat objectives, fixed and live-cell filter wheels, a camera (a pco.edge 4.2 sCMOS), and softWoRx imaging software (Leica Microsystems). Time-lapse imaging was performed in YE media in a CellASIC ONIX microfluidics perfusion system (Millipore Sigma). Cells were loaded into Y04C plates for 5 s at 8 psi , and YE liquid medium flowed into the chamber at 5 psi throughout the time-lapse. Images were acquired every 2 minutes with optical sections taken at 0.5 μm spacing. Time-lapse images were deconvolved with 10 iterations and visualized as maximum projections. Quantitative analysis of microscopy data was performed using Fiji (a version of ImageJ software available at https://fiji.sc) (Schindelin et al., 2012) . All quantifications were performed on non-deconvolved, sum projected images. For all intensity measurements, the background intensity was subtracted. The background intensity was found by taking a measurement in an area with no cells. The raw intensity of the background was divided by its area, which was multiplied by the area of the intensity measurement of interest. This number was subtracted from the raw integrated intensity of measurement of interest (Waters, 2009) . Data was graphed with Prism 8.0 (GraphPad Software).

## Reagents

**Table:** 

Strain	Genotype	Source
KGY246	*ade6-M210 leu1-32 ura4-D18* *h ^-^*	Lab stock
KGY481-2	*kin1 -mNG:hphMX6 mCherry-cdc15 sad1-mCherry :natMX6 ade6-M210 leu1-32 ura4-D18 h ^+^*	This study
KGY526-2	*pck1 -as2(M744G)-FLAG:kanMX6* *leu1-32 h ^+^*	Lab stock
KGY1405	*shk1 -as2(M460A):hphMX6 shk1 ::natMX6 ade6-M210 leu1-32 ura4-D18 h ^-^*	*(Loo and Balasubramanian, 2008)*
KGY1516	*kin1 -as1(F220G)-FLAG:kanMX6* *ade6-M210 leu1-32 ura4-D18 h ^+^*	*(Lee et al., 2018)*
KGY2711	* mid1::ura4 ^+^* *ade6-M210 leu1-32 ura4-D18h ^+^*	Lab stock
KGY3635-2	*shk1 -mNG:kanMX6 mCherry-cdc15 sad1-mCherry:natMX6 ade6-M210 leu1-32 ura4-D18 h ^-^*	This study
KGY3636-2	*pck1 -mNG:kanMX6 mCherry-cdc15 sad1-mCherry :natMX6 ade6-M210 leu1-32 ura4-D18 h ^-^*	This study
KGY4131-2	*pom1 -mNG:hphMX6 mCherry-cdc15 sad1-mCherry :natMX6 ade6-M210 leu1-32 ura4-D18 h ^+^*	This study
KGY4951-2	* mid1::ura4 ^+^ pom1 -as1(T778G)-tdTomato:natMX6 ade6-M210 leu1-32 ura4-D18h ^+^*	Lab stock
KGY5911-2	* mid1::ura4 ^+^ shk1 -as2(M460A):hphMX6 shk1 ::natMX6 ade6-M210 leu1-32 ura4-D18h ^-^*	This study
KGY19781	* mid1::ura4 ^+^ kin1 -as1(F220G)-FLAG:kanMX6* *ade6-M210 leu1-32 ura4-D18h ^+^*	This study
KGY19782	* mid1::ura4 ^+^ pck1 -as2(M744G)-FLAG:kanMX6* *ade6-M210 leu1-32 ura4-D18h ^+^*	This study
